# Schizotypal Traits in College Students: Association with Empathy and Psychiatric Symptoms

**DOI:** 10.7759/cureus.74995

**Published:** 2024-12-02

**Authors:** David Pérez-Ferrara, Yvonne Flores Medina, Guillermina Yáñez-Téllez, Rodolfo Solís-Vivanco, Alejandra Mondragón-Maya

**Affiliations:** 1 Iztacala Higher Education Faculty, Universidad Nacional Autónoma de México, Mexico City, MEX; 2 Clinical Research, Instituto Nacional de Psiquiatría Ramón de la Fuente Muñiz, Mexico City, MEX; 3 Laboratory of Cognitive and Clinical Neurophysiology, Instituto Nacional de Neurología y Neurocirugía "Manuel Velasco Suárez", Mexico City, MEX; 4 Faculty of Psychology, Universidad Nacional Autónoma de México, Mexico City, MEX

**Keywords:** affective empathy, cognitive empathy, psychiatric symptoms, schizotypal traits, young adults

## Abstract

Background: Recent research shows that individuals exhibiting schizotypal traits (ST) are more prone to developing other psychiatric disorders such as anxiety and depression. Regarding the relationship between empathy and schizotypy, a considerable degree of heterogeneity has been observed. The objective of this study was to describe the presence of ST in college students and the relationships among ST, psychiatric symptoms, and empathy.

Method: The present study employed a non-experimental, cross-sectional correlational design. A total of 70 participants were assessed with a semi-structured interview, the Oviedo Questionnaire for the Assessment of Schizotypy, the Symptom Checklist-90-Revised (SCL-90-R), and the Cognitive and Affective Empathy Test.

Results: A high prevalence of ST was identified in the study sample (85.8%), particularly regarding Distortion of reality (30%), Negative dimension (47%), and Interpersonal disorganization (70%). A positive correlation was observed between Distortion of reality and Interpersonal disorganization with most of the subscales of the SCL-90-R. A negative correlation was observed between Empathic joy and the Negative dimension of ST (rho (ρ) = -0.504, p = < .001), and a positive correlation between Empathic stress and Obsession-compulsion (ρ = 0.473, p < 0.001), Anxiety (ρ = 0.443, p < 0.001), and Depression (ρ = 0.368, p = 0.002). The results of the multiple linear regressions indicated that ST accounted for 41.8% of the variance in Obsession-compulsion, followed by Depression (33%), and Anxiety (27.2%). In addition, it was found that Empathic stress was a significant predictor of Anxiety (R² = 0.228, p ≤ .001), Obsession-compulsion (R² = 0.168, p ≤ .001), and Depressive symptoms (R² = 0.131, p = 0.002). Finally, it was observed that psychiatric symptoms fully mediated the effect of Empathic stress on Interpersonal disorganization and Distortion of reality.

Conclusions: There were distinctive patterns in which the different dimensions of ST are related to psychiatric symptoms and empathy.

## Introduction

Schizotypy is a condition that shares cognitive, behavioral, and emotional symptoms with schizophrenia, though it varies in intensity and duration. As a phenotype, schizotypal traits (ST) have been proposed to be expressed in the general population within the range of 5-8% [1]. Due to its similarity with schizophrenia, most studies focus on three symptomatic dimensions: positive (i.e., strange perceptual and hallucinatory experiences, and distortion of reality), negative (i.e., diminished experience of pleasure, a flattening of affect, and difficulties in interpersonal relationships), and disorganized (characterized by alterations in cognitive processes, language, and behavior) [2]. Also, similar to schizophrenia, ST increases the risk of deficits in neurocognitive domains (i.e., verbal memory, working memory, attention, cognitive flexibility) and social cognition (SC), as well as developing other psychiatric disorders, including anxiety and depression, or a psychotic disorder [3,4].

SC is an umbrella term that encompasses a set of processes associated with perceiving, encoding, and regulating emotional and social information from others and from ourselves [3]. In schizotypy, most SC studies have focused on emotion recognition [5] and theory of mind [6]. However, a smaller proportion of research has centered on empathy. Empathy is a complex and multidimensional concept. Despite the multitude of definitions that exist, there is a consensus among researchers that empathy can be divided into two broad categories: affective empathy and cognitive empathy [7]. Affective empathy has been defined as the capacity to experience an emotion generated by the emotional experience of the other. Affective empathy is a multifactorial process comprising three central dimensions: affective arousal, emotional contagion, and empathic concern [8]. Moreover, it is classified according to the valence of the emotions that are shared. This includes empathic stress, which refers to the capacity to empathize with others' negative emotions, and empathic joy, which denotes the ability to empathize with others' positive emotions [9]. In contrast, cognitive empathy has been defined as the capacity to discern and comprehend the emotional states of others. This aspect of empathy has been identified as more intricate and requires higher-order processes. Cognitive empathy includes perspective taking, a process closely related to the theory of mind, and emotional understanding, which involves the recognition and comprehension of emotional states [9]. With regard to the evolution of empathy and its constituent elements, there is evidence that affective empathy emerges prior to cognitive empathy, both at phylogenetic and ontogenetic levels [10]. In humans, the most fundamental components of affective empathy emerge during the first year of life. Affective arousal enables the automatic discrimination of stimuli (or their characteristics) as pleasant or unpleasant, threatening, or safe, and allows the organization of adaptive responses to such stimuli [8].

Findings from studies on empathy and schizotypy are inconsistent: some studies report positive correlations with ST positive dimension [11], while others report negative correlations with the three dimensions of schizotypy [12]. Also, affective empathy has shown inverse correlations with the negative dimension and positive correlations with positive and disorganized dimensions [12]. It is also noteworthy that there is a significant positive correlation between depressive symptoms and affective empathy [13]. Specifically, it has been suggested that affective empathy towards others' distress may be conceptualized as a "risk factor," whereby empathic responses to others' distress, if excessive, may contribute to the development of internalizing problems such as depression [14].

Due to these inconsistent results, the present study has two general objectives. The first one was to describe the presence of ST in college students and to explore the relationship between ST, psychiatric symptoms, and empathy. In this regard, it was expected that positive correlations would be found between ST and psychiatric symptoms. Furthermore, it was expected to observe a different pattern in the relationship between both dimensions of empathy and psychiatric symptoms, whereas cognitive empathy would exhibit inverse associations to psychiatric symptomatology, while affective empathy would be positively correlated. The second objective was to analyze the effect of ST on psychiatric symptoms as well as the effect of affective empathy on psychiatric symptoms, including ST. In this regard, it was expected that ST would act as a predictor of psychiatric symptoms. Additionally, due to the evidence about affective empathy as a risk factor for developing internalizing problems [14], it was hypothesized that affective empathy, particularly empathic stress, would act as a predictor of psychiatric symptoms, including, ST. The study of schizotypy in subclinical samples is a relevant avenue of research as it allows for the investigation of psychotic spectrum phenomena without the confounding effects commonly associated with clinical samples (e.g., medication, iatrogenic effects of psychotic breaks and hospitalization, social breakdowns, etc.) [15].

## Materials and methods

This was a non-randomized, cross-sectional study conducted in the Facultad de Estudios Superiores Iztacala (FESI) campus, Tlalnepantla de Baz, Mexico, which is part of the Universidad Nacional Autónoma de México (UNAM). The recruitment and assessment period was from April 3, 2023, to May 31, 2023.

Participation in the study was entirely voluntary, and all students included in the sample were required to sign an informed consent form. This study adhered to the tenets of the Declaration of Helsinki and was approved by the Ethics Committee of the Facultad de Estudios Superiores Iztacala (FESI) (registration number: CE/FES/2021/1384).

Sample size calculation

A non-randomized convenience sampling was used. A post hoc analysis was conducted to assess the statistical power of the sample obtained using the G*Power software. The lowest R² score obtained in the statistical analysis was used to calculate the power analysis, which yielded a power of 0.818, falling within the minimum expected parameters [16]. A total of 70 participants were recruited for this study.

Inclusion and exclusion criteria

To be included in the study, participants were required to be at least 18 years of age, regardless of gender, and be regular college students. Individuals with a current diagnosis of any psychiatric disorder or with self-reported perceptual impairments that might preclude assessment were excluded from the study.

Materials

Semi-Structured Interview

A semi-structured interview format was developed for the purpose of collecting socio-demographic data and verifying inclusion/exclusion criteria. This interview encompassed three primary areas: (1) family history of psychiatric and neurological disorders, (2) personal history of psychiatric and neurological disorders, and (3) medication and substance use. As this was an interview designed to obtain sociodemographic data, it was not possible to ascertain its validity or reliability.

Oviedo Questionnaire for the Assessment of Schizotypy (SCH-Q)

This instrument was designed to assess ST or psychotic tendencies in adolescents and young adults [2]. It is a self-report questionnaire that comprises 51 items, which are grouped into 10 subscales: Reference ideas, Magical thinking, Unusual perceptual experiences, Unusual thinking and language, Paranoid ideation, Physical anhedonia, Social anhedonia, Rare behavior, Lack of close friends, and Excessive social anxiety. Additionally, three dimensions can be obtained: Negative dimension, Distortion of reality, and Interpersonal disorganization. The scores are expressed in percentiles. Scores between 1 and 50 indicate the absence of symptoms, while scores between 51 and 80 are indicative of a moderate level of symptomatology, and scores above 81 are 10 subscales ranging from 0.68 to 0.90. The dimension exhibiting the lowest reliability was the Negative dimension, with a Cronbach’s alpha value of 0.67. This was followed by Interpersonal disorganization (0.83) and Distortion of reality (0.88), which demonstrated the highest reliability.

Symptom Checklist-90-Revised (SCL-90-R)

This self-report screening tool quantifies psychopathological symptoms that may indicate the presence of psychological distress or a psychiatric disorder [17]. The instrument was used to explore the presence of any potential psychiatric condition in the sample. It provides information clustered into ten psychiatric categories: Somatization, Obsession-compulsion, Interpersonal sensitivity, Depression, Anxiety, Hostility, Phobic anxiety, Paranoid ideation, Psychoticism, and Additional symptoms. A general score (GSI) can be obtained as well. In terms of the SCL-90-R's reliability in the Mexican population, it has been demonstrated that nine of the eleven dimensions exhibited satisfactory internal consistency values (Cronbach's alpha = 0.85). The overall Cronbach's alpha coefficient for the GSI was 0.96.

Cognitive and Affective Empathy Test (CAET)

This self-report instrument measures a global dimension of empathy and provides four empathy-related subscales besides the total score. The subscales associated with Cognitive empathy are Perspective taking, which denotes the capacity to adopt the perspective of another individual, and Emotional understanding, which is defined as the capacity to discern and comprehend the emotional states, intentions, and perceptions of others. The subscales associated with Affective empathy are Empathic stress which refers to the capacity to empathize with others' negative emotions; and Empathic joy, which denotes the ability to empathize with others' positive emotions [9]. This scale exhibits satisfactory reliability for the total scale (Cronbach's alpha = 0.86) and for the subscales of Perspective Adoption (Cronbach's alpha = 0.70), Emotional Understanding (Cronbach's alpha = 0.74), Empathic Stress (Cronbach's alpha = 0.78) and Empathic Joy (Cronbach's alpha = 0.75).

Procedure

The sample was recruited by social media (i.e., Facebook and Instagram; Meta Platforms, Inc., Menlo Park, California, United States) and print advertisements within FESI. It is important to note that the participants did not receive any form of economic compensation for their involvement in the study. Appointments were arranged on a face-to-face basis, contingent upon the availability of potential participants. At the appointment, the aims of the study were explained, and participants were required to sign the informed consent form. The evaluation was conducted in a quiet cubicle within the faculty, which was furnished with two desks and two swivel chairs. The semi-structured interview, SCL-90-R, CAET, and SCH-Q were administered afterward. The assessment session lasted approximately an hour.

Statistical analysis

Descriptive statistics were used for demographic data and SCH-Q scores. Spearman's rho (ρ) was used to explore the relationship between ST, psychiatric symptomatology, and empathy. A Holm-Bonferroni correction was applied due to the presence of multiple correlations, with significance established at a p-value of 0.003 or lower. Multiple linear regressions were performed to determine the effect of ST on psychiatric symptoms. Additionally, multiple linear regressions were used to determine the effect of Empathic stress on psychiatric symptoms and ST. Finally, mediation analyses were performed to identify the potential mediating effect of psychiatric symptoms in the relationship between Empathic stress and ST. For statistical analyses, JASP version 0.17.1.0 (JASP Team, University of Amsterdam, The Netherlands) was utilized. The absence of missing data in this study is a consequence of the joint efforts of two evaluators and the participants themselves. With regard to the outliers, no method was employed to eliminate them; consequently, all the data were included in the analyses.

## Results

The sample consisted of 70 Mexican college students (64% women), with a mean age of 18.36 years (SD = 0.59) and a mean education of 12.64 years (SD = 0.54). Regarding the SCL-90-R scores, heterogeneity was observed in the different scales. However, the means and medians were within the average ranges [16]. The mean of the GSI was 57.2 (SD = 9.71), while the median was 57.

Regarding the schizotypal dimensions, the highest mean was for Interpersonal disorganization (mean = 67.69, SD = 26.44), followed by Negative dimension (mean = 53.26, SD = 26.1), and Distortion of reality (mean = 39.1, SD = 27.58). Particularly, it was observed that 30% of the subjects exhibited moderate to severe symptoms of Distortion of reality, 48.6% of Negative dimension, and 70% of Interpersonal disorganization.

It is important to note that when participants were separated according to the presence, severity, and/or absence of symptoms, eight groups were obtained. The first group comprised participants who obtained percentile scores below 50 in all dimensions (No-ST). The following groups were formed according to the dimension(s) where they scored above 51 (Table 1). The extremes of the continuum were considered for comparisons, with the No-ST group and the DR-ND-ID group both comprising 10 participants each. The Mann-Whitney U test revealed significant differences between the groups in empathy, with the No-ST group exhibiting higher total CAET scores (U = 80, p = 0.025, r_b_ = 0.6) and higher scores on the subscales of Emotional understanding (U = 83, p = 0.013, r_b_ = 0.66), Perspective taking (U = 77.5, p = 0.039, r_b_ = 0.55) and Empathic joy (U = 85.5, p = 0.008, r_b_ = 0.71).

**Table 1:** Segmented groups according to the presence of moderate/severe symptoms in the SCH-Q dimensions. No-ST: no schizotypy traits, scores equal to or less than 50; DR: Distortion of reality; ID: Interpersonal disorganization; ND: Negative dimension; SCH-Q: Oviedo Questionnaire for the Assessment of Schizotypy

Group	Frequency	Percentage
No-ST	10	14.2
DR	2	2.8
ND	8	11.5
ID	16	22.8
DR+ND	1	1.5
DR+ID	8	11.5
ND+ID	15	21.5
DR+ND+ID	10	14.2

Correlations between ST, psychiatric symptoms, and empathy

Positive correlations were observed between Interpersonal disorganization and Distortion of reality with most of the subscales of the SCL-90-R. Interpersonal disorganization showed moderate to strong significant correlations with Phobic anxiety (ρ = 0.636, p < 0.001, Fisher’s z = 0.752), Depression (ρ = 0.591, p < 0.001, Fisher’s z = 0.679), Obsession-compulsion (ρ = 0.537, p < 0.001, Fisher’s z = 0.6), and Anxiety (ρ = 0.509, p < 0.001, Fisher’s z = 0.561) subscales. In contrast, Distortion of reality demonstrated moderate, statistically significant correlations with the subscales of Obsession-compulsion (ρ = 0.448, p < 0.001, Fisher’s z = 0.482), Somatization (ρ = 0.432, p < 0.001, Fisher’s z = 0.463), Depression (ρ = 0.363, p = 0.002, Fisher’s z = 0.380), and Anxiety (ρ = 0.348, p = 0.003, Fisher’s z = 0.363). Notably, the Negative dimension did not correlate with any SCL-90-R subscale.

In the case of empathy, the CAET total score did not show significant correlations with the SCL-90-R subscales. It is noteworthy that only the Empathic stress subscale exhibited significant positive correlations with the SCL-90-R: moderate positive correlations were observed for Obsession-compulsion (ρ = 0.473, p < 0.001, Fisher’s z = 0.514), Anxiety (ρ = 0.443, p < 0.001, Fisher’s z = 0.476), Interpersonal sensitivity (ρ = 0.395, p < 0.001, Fisher’s z = 0.418), and Depression (ρ = 0.368, p = 0.002, Fisher’s z = 0.386).

With regard to ST, the Negative dimension was negatively correlated with Empathic joy (ρ = -0.585, p = < .001, Fisher’s z = -0.669), and the total empathy score (ρ = -0.504, p = < .001, Fisher’s z = -0.554).

Multiple linear regression analyses: SCH-Q and SCL-90-R

Four multiple linear regression analyses were conducted, with the Negative dimension, Interpersonal disorganization, and Distortion of reality scores serving as the independent variables and the dependent variables being Depression, Anxiety, Obsession-compulsion subscales, and the GSI of the SCL-90-R. The effect of Negative dimension was considered but ultimately excluded from the analysis due to its lack of statistical significance. A p-value of 0.05 or higher was observed for all models in the Durbin-Watson test. The scale that exhibited a higher percentage of variance explanation was Obsession-compulsion (41.8%), followed by the total score (GSI, 38.6%), Depression (33%), and Anxiety (27.2%). It is noteworthy that the predictive capacity of the models was statistically significant (Table 2). We noted that Interpersonal disorganization constituted a greater factor within the equation for all models compared to Distortion of reality, and that the results indicated a positive correlation between the severity of Distortion of reality and Interpersonal disorganization symptoms and the SCL-90-R subscales (see Table 2).

**Table 2:** Multiple linear regressions SCL-90-R, SCH-Q. ^a^ p-value of the Durbin-Watson test greater than 0.05. Note. Negative dimension was considered as covariate but not included. SCL-90-R: Symptom Checklist-90-Revised; GSI: General Symptom Index of the SCL-90-R; ID: Interpersonal Disorganization subscale of the SCH-Q; DR: Distortion of reality subscale of the SCH-Q; SCH-Q: Oviedo Questionnaire for the Assessment of Schizotypy; ANX: Anxiety; DEP: Depression; OC: Obsession-compulsion

Model		R²	Adjusted R²	F Change	p^a^	β	t	p
OC		0.418	0.400	24.020	0.0001			
	DR					0.329	3.299	0.002
	ID					0.451	4.526	
DEP		0.330	0.310	16.528	0.0001			
	DR					0.222	2.071	0.042
	ID					0.457	4.278	
ANX		0.272	0.250	12.503	0.0001			
	DR					0.232	2.077	0.042
	ID					0.392	3.514	
GSI		0.386	0.368	11.228	0.001			
	DR					0.343	3.351	0.001
	ID					0.041	4.006	

Linear regression analyses: empathic stress, SCL-90-R, and SCH-Q

The effect of empathic stress on psychiatric symptoms, including ST, was evaluated through linear regressions. Empathic stress acted as the independent variable, while the dependent variables were the scores of the SCL-90-R subscales (i.e., Obsessive-compulsive, Depression, Anxiety, GSI) and the dimensions of the SCH-Q (i.e., Distortion of reality, Negative dimension, and Interpersonal disorganization). The highest effect of Empathic stress was observed on the Anxiety subscale (R^2^ = 0.228, p ≤ .001), followed by the GSI (R^2^ = 0.179, p ≤ .001), Obsessive-compulsive subscale (R^2^ = 0.168, p ≤ .001), and the Depression subscale (R^2^ = 0.131, p = 0.002). Regarding the effect of Empathic stress on ST, we observed a significant, but almost null effect on Interpersonal disorganization (R^2^ = 0.067, p = 0.03). It should be noted that the effect of Empathic stress on Distortion of reality and Negative dimension was not significant (Table 3).

**Table 3:** Linear regression analyses Empathic stress, SCL-90-R and SCH-Q ^a^ p-value of the Durbin-Watson test greater than 0.05. Note. Negative dimension was considered as covariate but not included. SCL-90-R: Symptom Checklist-90-Revised; GSI: General Symptom Index of the SCL-90-R; ID: Interpersonal disorganization subscale of the SCH-Q; DR: Distortion of reality subscale of the SCH-Q; SCH-Q: Oviedo Questionnaire for the Assessment of Schizotypy; ANX: Anxiety; DEP: Depression; OC: Obsession-compulsion; ND: Negative dimension

Model	R	R²	Adjusted R²	F Change	p^a^
OC	0.41	0.168	0.156	13.733	0.0001
DEP	0.362	0.131	0.119	10.279	0.002
ANX	0.478	0.228	0.217	20.085	0.0001
GSI	0.423	0.179	0.167	14.819	0.0001
DR	0.168	0.028	0.014	1.987	0.163
ND	0.078	0.006	-0.008	0.421	0.518
ID	0.259	0.067	0.053	4.891	0.03

**Figure 1:**
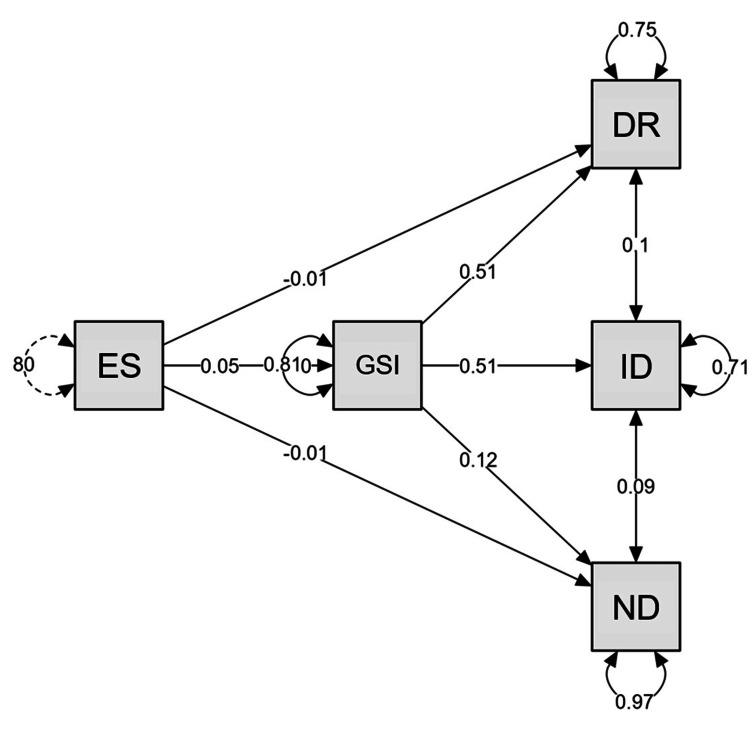
Mediation analysis – Path analysis ES: Empathic Stress subscale of CAET; GSI: General Symptom Index of the SCL-90-R; ID: Interpersonal Disorganization subscale of the SCH-Q; DR: Distortion of reality subscale of the SCH-Q; SCL-90-R: Symptom Checklist-90-Revised; SCH-Q: Oviedo Questionnaire for the Assessment of Schizotypy; CAET: Cognitive and Affective Empathy Test

As no significant effect of Empathic stress on schizotypy dimensions was identified, a mediation analysis was subsequently performed. The GSI of the SCL-90-R was selected as a mediating variable, given that it exhibited significant correlations with empathic stress (independent variable) and schizotypy dimensions (dependent variable). The results showed a non-significant direct effect between Empathic stress and Interpersonal disorganization (p = 0.711), Distortion of reality (p = 0.685), and Negative dimension (p = 0.321). However, when the GSI of the SCL-90-R is considered as a mediator, a significant positive effect of Empathic stress on Interpersonal disorganization (p = 0.003) and Distortion of reality (p = 0.003) was observed, although this effect was not observed for Negative dimension (p = 0.367). The results indicate a significant indirect effect, which can be considered a complete mediation, given that no significance was found in the direct effect. The results of the path analysis indicated that Empathic stress did not exert a direct influence on the various dimensions of ST, including Distortion of reality (β = -0.01), Interpersonal disorganization (β = 0.05), and Negative dimension (β = -0.01). However, the analysis revealed that Empathic stress did exert an indirect effect on Distortion of reality (β = 0.51) and Interpersonal disorganization (β = 0.51). It is noteworthy that no significant indirect effect was observed on Negative dimension (β = 0.12). These findings indicate that the mediating effect of psychiatric symptoms plays a pivotal role in the relationship between affective empathy and Interpersonal disorganization and Distortion of reality (Table 4, Figure 1).

**Table 4:** Mediation analysis Delta method standard errors, bias-corrected percentile bootstrap confidence intervals, ML estimator. ES: Empathic Stress subscale of CAET; ND: Negative Dimension; ID: Interpersonal Disorganization; DR: Distortion of Reality; GSI: General Symptom Index of the SCL-90-R; SCL-90-R: Symptom Checklist-90-Revised; CAET: Cognitive and Affective Empathy Test

	Estimate	Std. error	z-value	p-value	95% Confidence Interval	
					Lower	Upper
Direct effects						
ES → DR	-0.005	0.013	-0.406	0.685	-0.032	0.022
ES → ID	0.005	0.012	0.371	0.711	-0.025	0.032
ES → ND	-0.014	0.015	-0.992	0.321	-0.039	0.019
Indirect effects						
ES → GSI → DR	0.024	0.008	2.928	0.003	0.01	0.044
ES → GSI → ID	0.024	0.008	2.981	0.003	0.009	0.045
ES → GSI → ND	0.006	0.006	0.901	0.367	-0.008	0.02
Total effects						
ES → DR	0.019	0.013	1.43	0.153	-0.006	0.044
ES → ID	0.029	0.013	2.244	0.025	0.003	0.052
ES → ND	-0.009	0.013	-0.659	0.51	-0.034	0.018

## Discussion

The present study had two main objectives: first, to describe the levels of ST in a sample of college students and explore the relationship between ST, psychiatric symptoms, and empathy; and second, to examine the effects of ST on psychiatric symptoms; as well as the effect of affective empathy on psychiatric symptoms, including ST. This study has several notable strengths, including an in-depth examination of the various components of empathy and their correlation with ST and subclinical psychiatric symptoms in a sample of healthy subjects. It is noteworthy that, at the time of this study, no other studies were identified that had analyzed the relationship between these variables.

Presence of ST and associations between variables

With regard to the first objective, the results demonstrate that the university students assessed exhibited elevated features of schizotypy. The most prevalent features were those of Interpersonal disorganization (70%), followed by those of Negative dimension (48.6%) and Distortion of reality (30%). It is notable that only 14.3% of the sample exhibited no ST. This is not consistent with other studies, which report an ST presence range of 5-8% in the general population [1]. It is necessary to hypothesize that some factors may have contributed to the high percentages observed in the current sample.

The study was conducted a few months after the quarantine measures implemented during the COVID-19 pandemic were relaxed. This is pertinent given the heightened stress, anxiety, and depression levels that were reported in individuals as a consequence of concerns surrounding the novel coronavirus COVID-19 and the social isolation that was experienced as a result of the pandemic measures. In this context, Daimer et al. reported that concerns related to the impact of the COVID-19 pandemic were associated with higher levels of psychosis-related traits, anxiety, and depression [18]. Furthermore, the researchers concluded that social adversity predicted ST expression and that increased loneliness during the pandemic could accentuate ST as well. It is noteworthy that there is a consistent body of evidence indicating a relationship between loneliness and ST [19,20]. Furthermore, loneliness was identified as the primary risk factor for depression and anxiety during the COVID-19 pandemic [21]. Therefore, the pandemic may have contributed to an increase in the traits assessed in our sample, potentially due to an increase in loneliness, anxiety, stress, and depression. This is consistent with most existing literature which indicates a correlation between stress and ST. In a similar vein, Pignon et al. concluded that psychosocial stressors (i.e., traumatic events during childhood and stressful life situations) were associated with subclinical symptoms of psychosis [22]. Furthermore, they found that these stressors had independent effects on different dimensions of subclinical psychosis. This is consistent with the findings of Juan and Rosenfarb, who observed that significant stressful life events and daily difficulties were associated with an increased prevalence of schizotypal symptoms [23]. Finally, it is possible that inherent factors of the questionnaire may have influenced the results: for instance, as a self-report questionnaire, there is a possibility that it may have generated some measurement bias. In this context, discrepancies have been observed between the results of self-report questionnaires and clinical interviews designed to assess ST. Some studies have indicated that self-report questionnaires may overestimate the presence of symptoms when compared to clinical interviews [24].

Regarding the correlations, our findings indicated the existence of divergent patterns between Cognitive and Affective empathy in the context of the relationship between ST and psychiatric symptoms. The hypothesis in this regard was that a negative correlation would be observed between psychiatric symptoms, ST, and cognitive empathy. Nevertheless, the observed outcome was not consistent with such expectations. The analysis revealed no significant correlations between Cognitive empathy subscales and ST or SCL-90’s subscales. However, significant correlations between Affective empathy subscales and ST and psychiatric symptoms were found. This is consistent with the findings of Yan et al., who conducted a meta-analysis and found that Affective empathy, but not Cognitive empathy, was positively correlated with depressive symptoms, particularly in adolescence and young adulthood [13]. A noteworthy finding was the discovery of distinct patterns pertaining to the two components of Affective empathy: Empathic joy and Empathic stress. In this vein, the results demonstrated a notable negative correlation between the Empathic joy subscale and the Negative dimension of schizotypy. This suggests that individuals exhibiting greater negative ST demonstrate a diminished capacity to experience emotional contagion in response to others' positive emotions. This finding aligns with the observations reported by Stinson et al., who also identified negative correlations between Affective empathy measures and the Negative dimension of schizotypy [12]. Conversely, only Empathic stress was found to exhibit a significant positive correlation with the subscales of the SCL-90-R. These discrepancies in the correlations between Empathic joy and Empathic distress in relation to the other variables under investigation underscore the necessity for a differentiated approach to the analysis of Affective empathy. In this regard, numerous studies have identified distinct neural responses, in terms of intensity, to Empathic stress and Empathic joy [25].

Effect of ST on psychiatric symptoms

The second objective of this study was to ascertain the impact of ST on psychiatric symptoms. In this regard, it was hypothesized that ST dimensions would act as predictors of psychiatric symptoms. The results indicated that higher scores on the Interpersonal disorganization and Distortion of reality subscales were predictive of a greater number of psychiatric symptoms, thus supporting such a hypothesis. This is consistent with the findings of previous studies, which have reported positive correlations between ST and other psychiatric symptoms. In particular, positive correlations have been identified between depression, anxiety, suicidal behavior, and positive and negative affect [4,26].

It is noteworthy that certain studies have identified differences in the correlations across dimensions of schizotypy. The results indicated a higher correlation of psychiatric symptoms with Distortion of reality and Interpersonal disorganization, with no significant correlations observed with Negative dimension. This is consistent with the findings of Kwapil et al., who reported positive correlations between the Positive dimension and symptoms of depression, mania, and substance use, and did not find significant correlations with Negative dimension [26]. In a separate study, Kemp et al. reported that the Disorganization dimension was positively correlated with symptoms of depression, anxiety, social phobia, hypomania, and negative affect, while the Positive dimension was only positively associated with hypomania, and Negative dimension with depression and positive affect [27]. Conversely, Zhang et al. conducted a network analysis of ST and other subclinical psychiatric characteristics [28].

The study found that Interpersonal disorganization was the primary link between ST and autism. Additionally, the researchers reported that anxiety symptoms were only correlated with this dimension. The researchers concluded that the Positive and Interpersonal dimensions were the main nodes between schizotypal and obsessive-compulsive traits. Moreover, the study revealed a significant correlation between depression and all three dimensions of ST. These findings are consistent regarding the relationship between ST and psychiatric symptoms. Some mechanisms or hypotheses have been proposed to account for these relationships. One such hypothesis is associated with the hypothalamic-pituitary-adrenal (HPA) axis, which is a key axis in stress regulation and is involved in neurobiological processes found in different psychiatric disorders [1,29].

Furthermore, it is hypothesized that stability in Positive dimension traits could reinforce an alteration in the regulation of the mesolimbic dopamine system. In conjunction with high-risk contexts (e.g., bullying, loneliness, personality dimensions, genetics, etc.), this could increase the risk of developing a psychiatric disorder in adolescence [1,30]. Conversely, it has been documented that individuals with Interpersonal disorganization may experience difficulties in social interactions, a reduction in the number of friendships, a decline in self-confidence, and an increase in symptoms of social anxiety and depression due to their peculiar and unconventional behavior and speech [28]. Finally, it is important to note that there is considerable heterogeneity between the relationships of different dimensions of schizotypy and other psychiatric symptoms. This discrepancy may be attributed to differences in methodology, instruments, and even the studied populations.

Effect of empathic stress on ST and psychiatric symptoms

Our results indicated that Empathic stress was a significant predictor of Anxiety (22.8%), Obsessive-compulsive (16.8%), Depression (13.1%), and GSI symptoms (17.9%) in the model. It is important to note that due to certain limitations of the study, including its cross-sectional design and the characteristics of the sample (which was small and non-randomized), the observed effects in some variables should be interpreted with caution. In this regard, Tone and Tully proposed a model in which they posit that empathy may be a 'risky strength' due to the potential for empathic reactions to others' distress that are excessively aversive, involve excessive perspective-taking, or result in self-centered comforting responses or self-centered rumination about one's own role in the observed distress to facilitate the emergence of internalizing problems [14]. This is thought to occur through mechanisms such as personal distress and interpersonal guilt. Furthermore, inter-individual (e.g., peer relationships) and environmental factors (e.g., parenting styles) can impede the development of a typical empathic response. 

Given the absence of a relationship between ST and the Empathic stress subscale, an exploratory mediation analysis was conducted to ascertain the mediating effect of psychiatric symptoms on the relationship between Empathic stress and the three ST dimensions. The results indicated that psychiatric symptoms fully mediate the relationship between the Empathic stress subscale and the Distortion of reality and Interpersonal disorganization subscales. However, this was not the case for Negative dimension. In other words, the results indicated that individuals with high scores on Affective empathy, stressful emotional states, and the presence of psychiatric symptomatology were at an increased risk of presenting ST, particularly Interpersonal disorganization and Distortion of reality. This is consistent with the aforementioned model of empathy as a risk factor proposed by Tone and Tully [14]. Furthermore, the increase in these problems due to high Affective empathy scores may increase the probability of presenting vulnerability factors for schizotypy such as loneliness, isolation, and changes in neurotransmitter and hormonal systems [1,29,30]. It is crucial to highlight that, at the time of writing, no article was identified in the literature that reported and discussed the potential mediating role of psychiatric symptoms in the relationship between empathy and schizotypy. Consequently, it is recommended that future studies should undertake a more detailed analysis of these findings.

Limitations

It is important to note that the present study is subject to certain limitations. A non-randomized small sample size was assessed, and the study design was cross-sectional, which precludes the drawing of causal inferences between variables. Also, the sample consisted of young adults with a high level of education. Conversely, some variables that could be crucial in the reported relationships such as loneliness and stressful life events among others were not considered. Furthermore, the clinical measurements were obtained through self-report scales, so future studies should consider the use of more comprehensive clinical measurements that can corroborate the presence and severity of symptomatology. Similarly, empathy was also assessed by self-report, so it would be important for future studies to consider other more objective measures for the assessment of this construct.

## Conclusions

We found a higher percentage of ST than expected in our sample. This could be due to factors such as social isolation and affective changes as a result of the COVID-19 pandemic. We also found a significant effect of ST, especially Distortion of reality and Interpersonal disorganization, on psychiatric symptoms such as Anxiety, Depression, and Obsession-compulsion. Interestingly, we found no significant relationships between Cognitive empathy, ST, and psychiatric symptoms. Conversely, Affective empathy demonstrates significant relationships with ST and psychiatric symptoms. Particularly, we found that Empathic joy had a negative relationship with the Negative dimension of ST, and Empathic stress was positively correlated with psychiatric symptoms. Furthermore, we found significant effects of Empathic stress on the presence of Depression, Anxiety, and Obsessive-compulsive symptoms. Finally, we found that psychiatric symptoms acted as mediators in the relationship between Empathic stress and some dimensions of schizotypy, particularly Distortion of reality and Interpersonal disorganization.

This study underscores the significance of evaluating the interrelationship between diverse dimensions of empathy, psychiatric symptoms, and ST. Moreover, in the context of Affective empathy, it is crucial to distinguish between positive and negative valence emotions, as they appear to manifest disparate patterns. In light of the aforementioned findings, we propose that additional research investigate these distinct patterns of cognitive and affective empathy, with a particular focus on affective empathy and the associated differences in emotional valence. It is crucial to highlight that future research should prioritize longitudinal studies, which will facilitate a more robust assessment of the findings identified in this study and enable the utilization of clinical and objective measures to evaluate the variables under investigation.

## Appendices

Semi-structured interview

Date: _____/_____/_____

Name: _____________________________________________________________________

Age: ________             Sex: ________             Years of schooling: ________

Marital status: ___________________                          Occupation: ____________________   

Place of residence: _______________                           Phone number: ______________________

Family history of psychiatric and neurological disorders

Have any close relatives (parents or siblings) been diagnosed with any of the following diseases?

Schizophrenia: YES     NO

Bipolar Disorder: YES    NO

Depression: YES     NO

Anxiety: YES     NO

Epilepsy: YES     NO

Migraine: YES     NO

Dementia: YES     NO

Other: YES     NO

Specify: ______________________________________

Psychiatric and neurological history

Have you been diagnosed with any of the following diseases?

Epilepsy: YES     NO

ADHD: YES     NO

Migraine: YES     NO

Specific learning disabilities: YES     NO

TBI: YES     NO

Other: YES     NO 

Specify: ____________________________

Medications and drug use

Are you currently undergoing any traditional or alternative medical treatment?

YES     NO       Why? ___________________________________________________

Which medications do you use? ______________________________________________________________________________________________________________________________________________________________________________________________________________________________________________________________________

Do you smoke tobacco?            YES     NO

How much and how often? ___________________________________________________________

Do you consume alcohol?         YES     NO

How much and how often?  ___________________________________________________________

Do you use any of the following substances?

a. Marihuana/cannabis     YES     NO     Frequency: _______________________

b. Cocaine/crack     YES     NO     Frequency: _______________________

c. Heroin     YES     NO     Frequency: _______________________

d. Acids/LSD     YES     NO     Frequency: _______________________

e. Inhalants     YES     NO     Frequency: _______________________

f. Mushroom/peyote     YES     NO     Frequency: _______________________

g. Amphetamines     YES     NO     Frequency: _______________________

h. Others     YES     NO     Frequency: _______________________
